# Altered expression of Butyrophilin (*BTN*) and BTN‐like (*BTNL*) genes in intestinal inflammation and colon cancer

**DOI:** 10.1002/iid3.105

**Published:** 2016-04-01

**Authors:** Cristina Lebrero‐Fernández, Ulf Alexander Wenzel, Paulina Akeus, Ying Wang, Hans Strid, Magnus Simrén, Bengt Gustavsson, Lars G. Börjesson, Susanna L. Cardell, Lena Öhman, Marianne Quiding‐Järbrink, Anna Bas‐Forsberg

**Affiliations:** ^1^ Department of Microbiology and ImmunologyInstitute of BiomedicineUniversity of GothenburgGothenburgSweden; ^2^ Department of Internal Medicine and Clinical NutritionInstitute of MedicineUniversity of GothenburgGothenburgSweden; ^3^ Center for Functional GI and Motility DisordersUniversity of North CarolinaChapel HillNorth CarolinaUSA; ^4^ Department of SurgeryInstitute of Clinical SciencesUniversity of GothenburgGothenburgSweden; ^5^ School of Health and EducationUniversity of SkövdeSkövdeSweden

**Keywords:** Butyrophilin (Btn)‐like (Btnl), colon cancer, immune regulation, intestinal inflammation, irritable bowel syndrome (IBS), ulcerative colitis (UC)

## Abstract

Several Butyrophilin (BTN) and Btn‐like (BTNL) molecules control T lymphocyte responses, and are genetically associated with inflammatory disorders and cancer. In this study, we present a comprehensive expression analysis of human and murine *BTN* and *BTNL* genes in conditions associated with intestinal inflammation and cancer. Using real‐time PCR, expression of human *BTN* and *BTNL* genes was analyzed in samples from patients with ulcerative colitis, irritable bowel syndrome, and colon tumors. Expression of murine *Btn* and *Btnl* genes was examined in mouse models of spontaneous colitis (*Muc2* ^−/−^) and intestinal tumorigenesis (*Apc* ^Min/+^). Our analysis indicates a strong association of several of the human genes with ulcerative colitis and colon cancer; while especially *BTN1A1*, *BTN2A2*, *BTN3A3*, and *BTNL8* were significantly altered in inflammation, colonic tumors exhibited significantly decreased levels of *BTNL2*, *BTNL3*, *BTNL8*, and *BTNL9* as compared to unaffected tissue. Colonic inflammation in *Muc2* ^−/−^ mice significantly down‐regulated the expression of particularly *Btnl1*, *Btnl4*, and *Btnl6* mRNA, and intestinal polyps derived from *Apc* ^Min/+^ mice displayed altered levels of *Btn1a1*, *Btn2a2*, and *Btnl1* transcripts. Thus, our data present an association of *BTN* and *BTNL* genes with intestinal inflammation and cancer and represent a valuable resource for further studies of this gene family.

## Introduction

Butyrophilin (BTN) and butyrophilin‐like (BTNL) proteins share significant homology and structural features with B7‐molecules and like B7‐molecules consist of regulatory molecules that modulate T‐cell mediated immune responses 1, 2, 3, 4, 5, 6, 7, 8, 9, 10, 11. Although T‐cell regulation by BTN and BTNL proteins is now unfolding, little is still known about the proteins' role in inflammation and proliferative disorders. Polymorphism in the human *BTNL2* gene has been linked to inflammatory disorders such as sarcoidosis, ulcerative colitis (UC), rheumatoid arthritis and myositis 12, 13, 14, 15, 16, and to prostate cancer 17. Furthermore, over‐expression of Btnl2 has been reported in *Mdr1a* ^−/−^ mice, a mouse model of intestinal bowel disease 2, and Btn2a2 deficiency was recently described to potentiate anti‐tumor responses 10. Moreover, a few studies have identified an association between human BTN3 and ovarian cancer 5, 18, 19. As several of human and murine *BTN* and *BTNL* genes are expressed in the intestine, their regulation may be relevant for gastrointestinal disorders. In order to determine how *BTN* and *BTNL* genes are regulated in intestinal inflammation and tumors, we used real‐time PCR to map the expression of human *BTN* and *BTNL* genes in patients with UC, irritable bowel syndrome (IBS) and colon cancer, and analyzed the presence of murine *Btn* and *Btnl* genes in mucin deficient mice (*Muc2* ^−/−^), a mouse model that reflects clinical and cellular features of human ulcerative colitis 20, 21, and in *Apc* ^Min/+^ mice, a spontaneous mouse model of intestinal cancerogenesis 22.

## Results and Discussion

### Expression of human and murine *BTN* and *BTNL* RNA in normal colon

**Figure 1:**
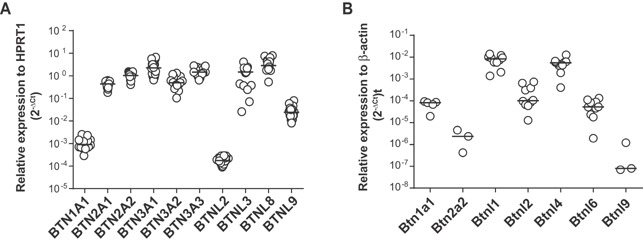
Expression of *BTN* and *BTNL* genes in human (A) and mouse (B) colon. Real‐time PCR analysis was conducted in colon biopsies of healthy subjects (*n* = 18) (A), and colonic tissue from C57BL/6 mice (*n* = 10) (B). The expression of *Btn1a1*, *Btn2a2*, and *Btnl9* was below the limit of detection for some of the animals. Each qPCR analysis was run in duplicate. Results were analyzed using the 2^−ΔCt^ method with *HPRT1* (A) and *β‐actin* (B) as a reference gene. Symbols represent individual values and horizontal lines the median.

Using real‐time PCR we examined the expression of human *BTN* and *BTNL* genes in normal colon. The level of expression varied considerably with relatively high expression of *BTN2A1*, *BTN2A2*, *BTN3A1*, *BTN3A2*, *BTN3A3*, *BTNL3, and BTNL8* genes, and low levels of *BTN1A1*, *BTNL2*, and *BTNL9* mRNA (Figure 1A). Variable *Btn* and *Btnl* gene expression was also identified in the murine colon; while *Btnl1* and *Btnl4* genes showed relatively high expression levels, *Btn2a2* and *Btnl9* transcripts were on the limit of detection (Figure 1B). An extended analysis of *Btnl9* mRNA expression in a panel of tissues demonstrated low expression in murine mesenteric lymph nodes, thymus, spleen and liver, and levels below the limit of detection in small intestine (Supporting Information Figure S1).

### Altered expression of human *BTN* and *BTNL* genes in UC and colon cancer indicates a role in dampening of intestinal inflammation and tumor immune surveillance

**Figure 2:**
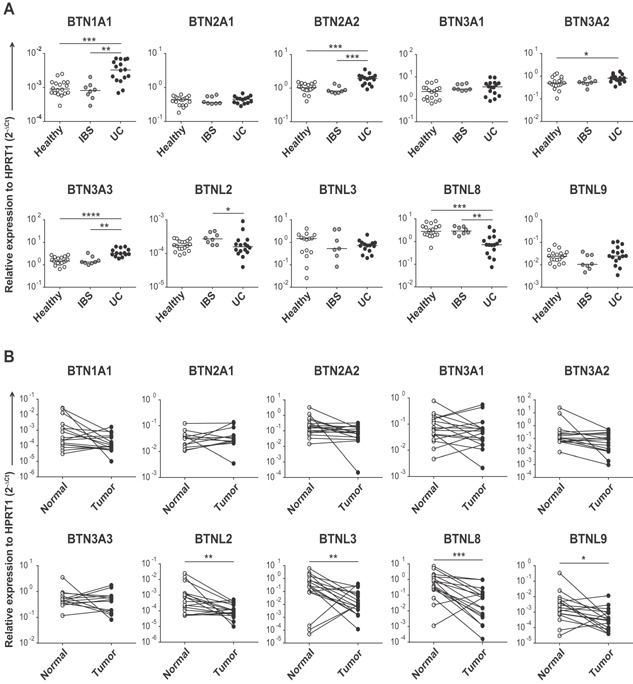
Expression of human *BTN* and *BTNL* genes in intestinal inflammation and colon cancer. (A) Gene expression in total RNA derived from colon of healthy individuals (*n* = 18), patients with IBS (*n* = 8) and UC (*n* = 16) was assessed by qPCR, run in duplicates, and determined using the 2^−ΔCt^ method with *HPRT1* as a reference gene. Statistical significance between groups was assessed using Kruskal–Wallis test followed by Dunn's multiple comparison test (*P ≤ 0.05, **P ≤ 0.01, ***P ≤ 0.001, and ****P ≤ 0.0001). Symbols represent individual values and horizontal lines the median. (B) Gene expression in tumor tissue and unaffected tissue from colon cancer patients (*n* = 17) was analyzed by qPCR, run in duplicates, and determined using the 2^−ΔCt^ method with *HPRT1* as a reference gene. Wilcoxon matched‐pairs signed rank test was used for statistical analysis (*P ≤ 0.05, **P ≤ 0.01, ***P ≤ 0.001, and ****P ≤ 0.0001). Connecting lines show values from samples taken from the same individual.

To assess *BTN* and *BTNL* genes' regulation in gastrointestinal disorders, we analyzed their expression in colon tissue from UC and IBS patients, and compared to the expression in healthy subjects with no prior history of gastrointestinal disorders. Our data showed a significant upregulation of *BTN1A1*, *BTN2A2*, *BTN3A2*, and *BTN3A3* genes in UC patients compared to healthy controls (Figure 2A, Table 1). In contrast, the expression of most of the *BTNL* genes was unchanged; only *BTNL8* was substantially altered in UC colon displaying significantly decreased mRNA levels (Figure 2A, Table 1). This suggests that the reported BTNL2 single nucleotide polymorphisms (SNPs) associated with UC 13 most likely affect the biological property of the encoded protein, as in the case of sarcoidosis 12, rather than gene expression level. The contrasting pattern of regulation of the *BTN* genes and *BTNL8* in UC is intriguing as human BTN3 and BTNL8 reportedly possess divergent functions in their capacity to stimulate peripheral T cells: while BTN3 seems to suppress T‐cell proliferation and cytokine secretion 5, 7, BTNL8 has been demonstrated to augment activation of T cells 4. If BTN3 and BTNL8 exhibit similar functions in the gut mucosa, upregulation of BTN3 and downregulation of BTNL8 would result in the same scenario, namely inflammation induced suppression of T‐cell mediated immune responses and may represent a feedback mechanism to limit the ongoing inflammation. Previous studies report increased expression of pro‐inflammatory cytokines such as IL‐6 and IFN‐γ in UC as compared to healthy controls 23. Examining the association between the regulation of *BTN* and *BTNL8* genes and the elevated levels of *IL6* and *IFNγ* RNA revealed an inverse correlation between *IFNγ* and *BTN3A3* (Supporting Information Figure S2) but no correlation between the pro‐inflammatory cytokines and the *BTN1A1*, *BTN2A2*, *BTN3A2* or *BTNL8* genes (data not shown). The association between the increased expression of *BTN3A3* and decreasing *IFNγ* levels, as well as recent data that provide evidence that murine Btn2a2 is a co‐inhibitory molecule that negatively modulates T‐cell mediated immune responses 10, suggests that BTN molecules indeed may represent a feedback mechanism counteracting the effect of inflammation. Although a powerful immune response may be host‐protective, a tight regulation of the intestinal *BTN* and *BTNL* genes may be important for attenuating T‐cell mediated immune responses and thus for limiting tissue damage and progression to chronic inflammation. To our knowledge, the immune regulatory capacity of human and murine BTN1A1 has yet not been characterized, and thus, the consequence of an over‐expression of this gene in conditions associated with inflammation remains to be investigated. In addition to patients with UC, we also analyzed colonic samples from patients with IBS. As shown in Figure 2A, our data demonstrated unchanged levels of both *BTN* and *BTNL* mRNA. Thus, normal *BTN* and *BTNL* gene expression in IBS, a condition without macroscopic signs of intestinal inflammation, but altered gene expression in inflamed UC colon, suggests inflammation‐driven *BTN* and *BTNL* gene regulation.

**Table 1:** Summary of gene expression data

	UC patients			Colon cancer patients
*BTN1A1*	↑↑↑			↔
*BTN2A1*	↔			↔
*BTN2A2*	↑↑↑			↔
*BTN3A1*	↔			↔
*BTN3A2*	↑			↔
*BTN3A3*	↑↑↑↑			↔
*BTNL2*	↔			↓↓
*BTNL3*	↔			↓↓
*BTNL8*	↓↓↓			↓↓↓
*BTNL9*	↔			↓
	*Muc2* ^−/−^ mice			
	Proximal	Middle	Distal	*Apc* ^Min/+^ mice TUM
*Btn1a1*	↔	↔	↓	↑
*Btn2a2*	↔	↔	↔	↑↑
*Btnl1*	↔	↓↓↓	↓↓↓↓	↓
*Btnl2*	↔	↔	↔	↔
*Btnl4*	↔	↓	↓↓	↔
*Btnl6*	↔	↓	↓↓	↔
*Btnl9*	↔	↔	↓	ND

Gene expression in UC and colon cancer patients as compared to healthy controls or adjacent unaffected tissue, respectively. Gene expression in *Muc2* ^−/−^ and small intestinal polyps derived from *Apc* ^Min/+^ mice as compared to *Muc2* ^+/−^ or adjacent unaffected tissue, respectively. ↑ indicates significant up‐regulation (↑ *P* ≤ 0.05, ↑↑ *P* ≤ 0.01, ↑↑↑ *P* ≤ 0.001, and ↑↑↑↑ *P* ≤ 0.0001). ↓ indicates significant down‐regulation (↓ P ≤ 0.05, ↓↓ P ≤ 0.01, ↓↓↓ P ≤ 0.001 and ↓↓↓↓ P ≤ 0.0001). ↔ no significant up‐ or down‐regulation. ND: not detectable.

SNPs in BTNL2 have been reported to be associated with increased susceptibility to prostate cancer 17, and high BTN3 expression levels in ovarian cancer has been suggested to contribute to immune evasion by damping the activity of infiltrating T cells 5. To elucidate the importance of BTN and BTNL mediated immune regulation in colon cancer we investigated mRNA expression of these genes in tumor tissue and in adjacent unaffected tissue from the same individuals. The analysis revealed unaffected *BTN* levels but a significant downregulation of *BTNL2*, *BTNL3*, *BTNL8*, and *BTNL9* mRNA in the colon tumors (Figure 2B, Table 1). Although only a few studies have addressed the function of the human BTNL proteins and hence their role in health and disease is still not well documented, it is plausible to speculate that a downregulation of *BTNL* genes in the tumor may be an additional way of tumors to modulate tumor‐specific T‐cell responses, especially as BTN and BTNL molecules have the ability to control both αβ and γδ T‐cell mediated responses 1, 2, 3, 4, 5, 6, 7, 8, 9, 10, 11, 24, 25, 26. Indeed, in view of recent studies demonstrating the capacity of human Vδ1 γδ T cells to kill colonic cancer cells 27, 28, and γδT17 cells to promote colorectal cancer progression by secreting IL‐17 29, an altered tumor‐associated expression of *BTNL* genes may have implications in the immune surveillance against colonic tumors. To determine whether the altered expression of the *BTNL2*, *BTNL3*, *BTNL8*, and *BTNL9* genes was associated with expression of pro‐inflammatory cytokines, we examined the expression of IL‐6 and IFN‐γ in the colon tumors. In contrast to *IFNγ* RNA that showed similar levels of expression in colonic tumors and adjacent unaffected tissue (data not shown), the expression of *IL6* RNA was significantly upregulated in the tumors (Supporting Information Figure S3). The increased level of *IL6* transcripts, however, did not correlate with the decreased level of the *BTNL* genes (data not shown).

### *Btn* and *Btnl* genes are regulated in murine models of spontaneous colitis and tumorigenesis

**Figure 3:**
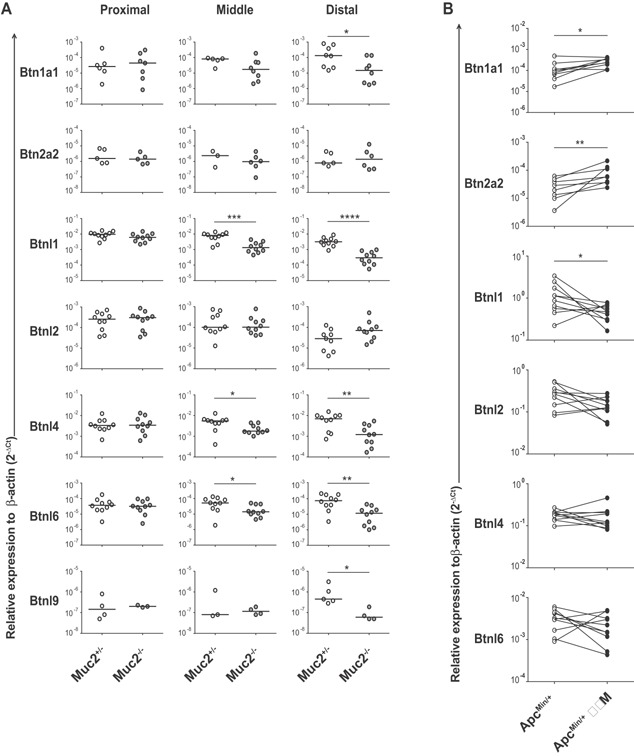
Expression of murine *Btn* and *Btnl* genes in intestinal inflammation and cancer. (A) Expression of *Btn* and *Btnl* genes in proximal, middle and distal colon sections of *Muc2* ^+/−^ (*n* = 10) and *Muc2* ^−/−^ (*n* = 10) mice was analyzed by qPCR, run in duplicates, and results were normalized to *β‐actin*. The expression of *Btn1a1*, *Btn2a2*, and *Btnl9* was below the limit of detection for some of the animals. Statistical significance was assessed using the Mann–Whitney test (*P ≤ 0.05, **P ≤ 0.01, ***P ≤ 0.001, and ****P ≤ 0.0001). Symbols represent individual values and horizontal lines the median. (B) Expression of *Btn* and *Btnl* genes was examined in intestinal polyps (TUM) and adjacent unaffected intestinal tissue of *Apc* ^Min/+^ mice (*n* = 10) by qPCR, run in duplicates, and normalized against *β‐actin*. Wilcoxon matched‐pairs signed rank test was used for statistical analysis (*P ≤ 0.05, **P ≤ 0.01, ***P ≤ 0.001, and ****P ≤ 0.0001). Connecting lines show values from samples taken from the same mouse.

The advent of genetic manipulation has moved mouse models of human disease, such as inflammatory bowel disease (IBD) and intestinal cancer, to the forefront. To better understand the role of Btn and Btnl family members in intestinal stress, *Btn* and *Btnl* genes were assessed for expression in *Apc* ^Min/+^ mice and in *Muc2* ^−/−^ animals. Similar to UC, inflammation in *Muc2* ^−/−^ mice results in loss of colon architecture and increases proximally from the distal part of the colon where the inflammation starts and is most prominent 20, 21, 30, 31. We compared the expression of *Btn* and *Btnl* genes in distal, middle, and proximal colon of *Muc2* ^−/−^ animals, and *Muc2* ^+/−^ controls without signs of inflammation. The analysis revealed a significant downregulation of *Btn1a1*, *Btnl1*, *Btnl4*, *Btnl6*, and *Btnl9* genes in the distal part of the large intestine of *Muc2* ^−/−^ animals, but no difference in levels of expression in the proximal, less affected part of the colon, suggesting inflammation‐related regulation of these genes (Figure 3A, Table 1). The observation that downregulation of *Btnl1*, *Btnl4*, and *Btnl6* expression associated with inflammation was particularly intriguing as these genes are essentially restricted to intestinal epithelia 9. We previously reported that Btnl1 attenuates the epithelial response to activated TCRαβ^+^ and TCRγδ^+^ intraepithelial T lymphocytes (IELs), resulting in reduced production of pro‐inflammatory mediators such as IL‐6 and CXCL1 9. Furthermore, our recent data show that Btnl1 and Btnl6 have the capacity to promote IEL proliferation 11. As intraepithelial γδ TCR IELs contribute to epithelial cell growth and differentiation 32, a reduced expression of mucosal *Btnl* genes may not only result in higher levels of IL‐6 and CXCL1, which are major contributors to intestinal immune pathology by promoting influx of monocytes and neutrophils respectively 33, 34, but may also impair epithelial cell regeneration and tissue repair. Hence, under‐expression of intestine specific Btnl molecules may have consequences for the control of tissue destructive inflammation and may contribute to the progression to chronic inflammation. Although our and others recent investigations have provided growing evidence for immune regulation by Btnl1 and Btnl6 3, 9, 11 the function of Btnl4 and Btnl9 remain to be defined.

In contrast to the reported overexpression of *Btnl2* RNA in *Mdr1a* ^−/−^ mice 2, we observed no significant change in *Btnl2* mRNA expression in *Muc2* ^−/−^ animals. This discrepancy may be explained by genetic background differences between the FVB *Mdr1a* ^−/−^ and C57BL/6 *Muc2* ^−/−^ mice, but may also result from the distinct molecular deficiencies initiating disease in the two models or from differences in the microbiota known to drive the inflammation in at least the *Muc2* ^−/−^ model. With regard to Btnl2, the *Muc2* ^−/−^ mice seem to be more similar to human UC as has been observed also in other studies 31.

Analysis of *Btn* and *Btnl* genes in *Apc* ^Min/+^ mice revealed increased levels of *Btn1a1* and *Btn2a2* genes in isolated small intestinal polyps as compared to adjacent unaffected tissue (Figure 3B, Table 1). In contrast, the polyps displayed unchanged *Btnl* mRNA levels, with the exception of *Btnl1* that showed a slight, yet significant decrease as compared to healthy controls. High expression of Btn1a1 and Btn2a2, both reported to inhibit activation of T cells 6, 10, 35, in intestinal tumors may contribute to immune evasion by damping the activity of infiltrating T cells. This is consistent with a recent study reporting impaired tumor growth and increased tumor infiltration by immune cells in *Btn2a2* ^−/−^ mice indicating a potentiated anti‐tumor response in the absence of Btn2a2 10. Likewise, attenuated levels of Btnl1 that, as discussed earlier, attenuates the epithelial response to activated intestinal IELs and promotes IEL proliferation, may lead to increased levels of IL‐6 which in a chronic phase of inflammation can contribute to colitis associated cancer by enhancing the proliferation and survival of tumor‐initiating intestinal epithelial cells 36. Moreover, γδ TCR IELs exhibit lytic activity against transformed epithelial cells 37, 38, 39, and thus reduced levels of IELs, as a consequence of attenuated Btnl1, may further promote tumor development.

## Concluding Remarks

Although previous studies report an association of BTN and BTNL with inflammation and cancer 5, 12, 13, 14, 15, 16, 17, 18, this is to our knowledge the first study that provides a comprehensive expression analysis of these genes in intestinal inflammation and colon cancer. Although only Btn1a1, Btn2a2, Btnl2, and Btnl9 are clear orthologs of human BTN and BTNL molecules 40, broad functional potentials may be conserved across the murine and human BTN and BTNL family. Indeed, even though particularly the orthologs Btn1a1 and Btn2a2 appeared to be differently regulated during inflammatory stress across the two species as evident from the divergence in expression in intestinal pathology, inflammation as well as carcinogenic stress consistently down‐regulated the expression of *BTNL* genes both in human and in mouse suggesting conserved inter‐species regulation of these genes. Although further analysis will be necessary to evaluate the consequence of the regulation of the *BTN* and *BTNL* genes and to clarify if the different regulation is a cause or effect of inflammation, our data clearly add strength to the evidence of a link between BTN and BTNL family members and inappropriate T‐cell activation and inflammation. In summary, identification of tumor and UC specific genetic regulation is necessary to understand the forces driving development of these diseases and in the longer run identify targets for diagnosis and therapeutics. Our results identify a substantial and significant modulation of several of human and murine *BTN* and *BTNL* genes in colonic inflammation and intestinal tumors, and thus present several BTN and BTNL family members to further investigate for UC and colon cancer susceptibility.

## Materials and Methods

### Patients and specimen collection

Sixteen patients with UC were included at the time of diagnosis (6 males and 10 females, aged 19–66, median age 42). All patients had an active extensive colitis with Mayo score 5–11, median 9. Patients were recruited at the out‐ and inpatient clinics, and the endoscopy units at Sahlgrenska University Hospital, Gothenburg, and Södra Älvsborgs Hospital, Borås, Sweden. None of the UC patients had other severe diseases such as heart, lung or neurological disease, or active malignancies. Biopsies were taken from a narrow inflamed area in the rectum. Further, 8 patients with IBS were included (all females, aged 22–60, median age 38). The patients were recruited at the outpatient clinic at Sahlgrenska University Hospital, Gothenburg, Sweden and met the Rome III criteria for diarrhea‐IBS 41. Exclusion factors included collagenous and lymphocytic colitis based on standard criteria, known celiac disease or other food allergies. Routine histology defined biopsies as non‐inflammatory. Additionally, 18 healthy subjects with no prior history of gastrointestinal disorders or bowel symptoms occurring within the prior seven days were included as controls in this study (1 male and 17 females, aged 23–48, median age 29). Sigmoidal colon biopsies were obtained 25–35 cm proximal from the anus of IBS and healthy controls during an unprepared sigmoidoscopy. Intestinal biopsies during colonoscopy/sigmoidoscopy were placed in RNAlater (Ambion^®^, Foster City, CA) for 24 h before freezing at −80°C and subsequent RNA extraction.

Seventeen individuals undergoing curative resection of colon tumors at the Sahlgrenska University Hospital were included in the studies (10 males and 7 females, aged 42–79, median age 63). Additional patient data is presented in Supporting Information Table S1. None of the cancer patients suffered from autoimmune disease or had undergone radiotherapy or chemotherapy for at least three years prior to colectomy. Immediately after colectomy, biopsies were collected from the tumor tissue and from unaffected mucosa located at least ten centimeters away from the tumor and placed in RNAlater (Ambion^®^, Foster City, CA) for 24 h before freezing at −80°C and subsequent RNA extraction.

### Ethical statement

This study was performed according to the Declaration of Helsinki and approved by the Regional Ethical Review Board in Gothenburg. All volunteers gave a written informed consent before participation.

### Mice

*Muc2* ^−/−^ mice on the C57BL/6 background were bred as *Muc2* ^−/−^ ×* Muc2* ^+/−^ at the University of Gothenburg. Protocols were approved by the Gothenburg animal ethics committee (Göteborgs djurförsöksetiska nämnd; permits 310–2010 and 280–2012), and institutional animal use and care guidelines were followed.

The *Apc* ^Min/+^ mutation occurred and is maintained on the C57BL/6 genetic background. The *Apc* ^Min/+^ breeding was maintained by crossing male *Apc* ^Min/+^ mice with female *Apc^+/+^* mice. Both male and female mice from this breeding were used for experiments. All mice were bred and maintained at the department of Experimental Biomedicine, University of Gothenburg. Animal experiments in this study were approved by the animal ethics committee in Gothenburg (Göteborgs djurförsöksetiska nämnd; permit 110–2013).

### Preparation of mouse tissues

*Muc2* ^−/−^ and *Muc2* ^+/−^ mice were sacrificed at 8–24 weeks of age. The colon was loosened from fat and luminal contents were removed. The colon was opened longitudinally, rinsed with phosphate buffered saline (PBS), divided into proximal, middle and distal colon, and saved in RNA later (Qiagen, Valencia, CA).

*Apc* ^Min/+^ mice were sacrificed at 15 weeks of age. The small intestines were flushed with PBS from both sides using blunt end gavage needles to remove fecal material, and cut open longitudinally. Small intestinal polyps and adjacent unaffected tissue were collected and saved in RNA later (Qiagen, Valencia, CA).

### RNA extraction and cDNA preparation

Human and murine tissue was lysed and homogenized (TissuelyserII, Qiagen, Valencia, CA) and total RNA was isolated using RNeasy^®^ mini kit (Qiagen, Valencia, CA), including DNAse I digestion. Human specimen was treated with Qiashredder (Qiagen, Valencia, CA) before RNA isolation. RNA concentration was determined spectrophotometrically (NanoDrop ND‐1000). The Omniscript kit (Qiagen, Valencia, CA) was used for cDNA synthesis, using 2000 ng RNA as template in a total reaction volume of 20 μL for human samples, and SuperScript^TM^ III Reverse Transcriptase kit (Invitrogen^TM^, Life Technologies, Carlsbad, CA) was used for cDNA synthesis, using 1000 ng RNA as template in total reaction volume of 20 μL for murine samples.

### Quatitative real‐time PCR

Expression of human and murine *BTN* and *BTNL* complementary DNA (cDNA) as well as human *IL6* cDNA was measured by quantitative real‐time PCR using GoTaq^®^ qPCR Master Mix, according to manufacturer's instructions (Promega, Madison, WI). The qPCR was performed on a LightCycler480 thermal cycler (Roche, Mannheim, Germany). Btn and Btnl PCR primers, listed in Supporting Information Table S2, span exon–exon borders to avoid amplification of genomic DNA. Primers for detection of *IL6* mRNA were purchased from Applied Biosystems (Hs00985639_m1; Foster City, CA). RNA expression was normalized to the expression of a housekeeping gene: human *HPRT1* or murine *β‐actin*. Each qPCR analysis was run in duplicate.

### Statistical analysis

Data were generated using GraphPad Prism version 6.04. The unpaired 2‐tailed nonparametric Mann–Whitney test was used for comparison between two independent groups, while Kruskal‐Wallis test followed by Dunn's multiple comparison was applied to evaluate differences between three groups. Statistical significance between two paired groups was determined by Wilcoxon matched‐pairs signed rank test. Differences were considered as statistically significant when *P* < 0.05 (*P ≤ 0.05, **P ≤ 0.01, ***P ≤ 0.001, and ****P ≤ 0.0001).

## Author Contribution

C.L‐F. performed experiments, analyzed data, prepared figures for the manuscript and contributed to manuscript writing; U.A.W. performed experiments and analyzed data; P. A. and Y.W. performed experiments; H.S., M.S., B.G., and L.G.B. provided samples of human biological material; S.L.C. contributed to experimental design and manuscript writing; M.Q‐J. and L.Ö. contributed to experimental design, interpreted the data and contributed to manuscript writing; A.B‐F. designed and supervised the project, interpreted the data and wrote the paper. All authors approved the final version.

## Conflict of Interest

None declared.

## Supporting information

Additional supporting information may be found in the online version of this article at the publisher's web‐site.

**Figure S1**. *Btnl9* expression.
**Figure S2**. *BTN3A3* expression inversely correlates with the expression of IFNγ in colon tissue from UC patients.
**Figure S3**. Expression of human *IL6* in colon cancer.

**Table S1**. Characteristics of the colon cancer patients included in the study.

**Table S2**. Primer sequences.
