# Supplementary material for: Altered expression of Butyrophilin (BTN) and BTN‐like (BTNL) genes in intestinal inflammation and colon cancer
Source: Immun Inflamm Dis. 2016 Apr 1;4(2):191–200. doi: 10.1002/iid3.105 (PMC4879465; doi:10.1002/iid3.105)
Supplement: Supplementary file 1 — Figure S1. Btnl9 expression. Figure S2. BTN3A3 expression inversely correlates with the expression of IFNγ in colon tissue from UC patients. Figure S3. Expression of human IL6 in colon cancer. [file IID3-4-191-s001.pdf]

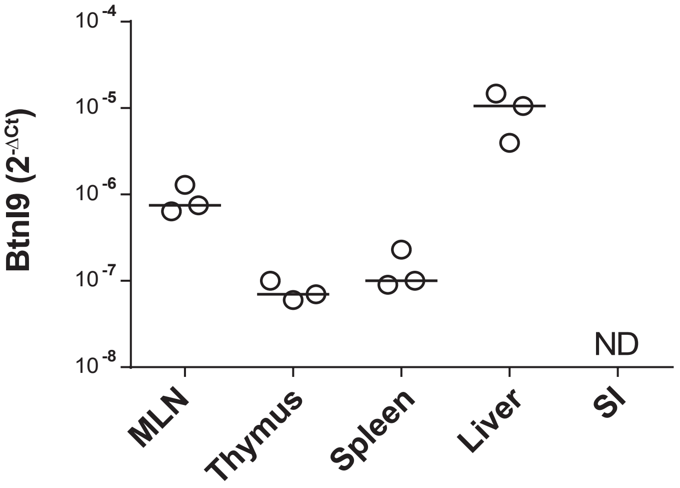

Supporting Information Figure 1. *Btnl9* expression. Tissues from C57BL/6 mice were analyzed for *Btnl9* expression by qPCR. Gene expression was determined using $2^{-\Delta Ct}$ method with β-*actin* as the reference gene. Data from 3 mice run in duplicates are shown. Symbols represent individual values and horizontal lines the median. MLN: mesenteric lymph nodes; SI: small intestine; ND: not detectable.

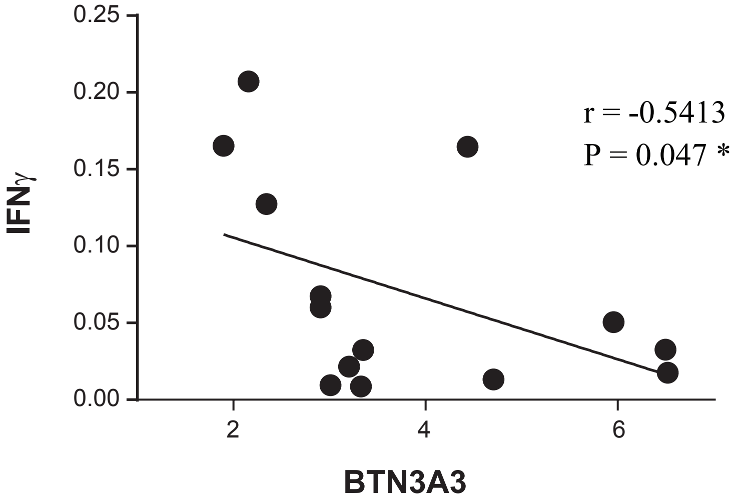

Supporting Information Figure 2. *BTN3A3* expression inversely correlates with the expression of *IFNγ* in colon tissue from UC patients. Gene expression was analyzed by qPCR and determined using the $2^{-\Delta Ct}$ method with *HPRT1* as the reference gene. Correlation between *BTN3A3* and *IFNγ* expression was assessed using the Spearman correlation test (*$P \leq 0.05$, **$P \leq 0.01$, ***$P \leq 0.001$ and ****$P \leq 0.0001$).

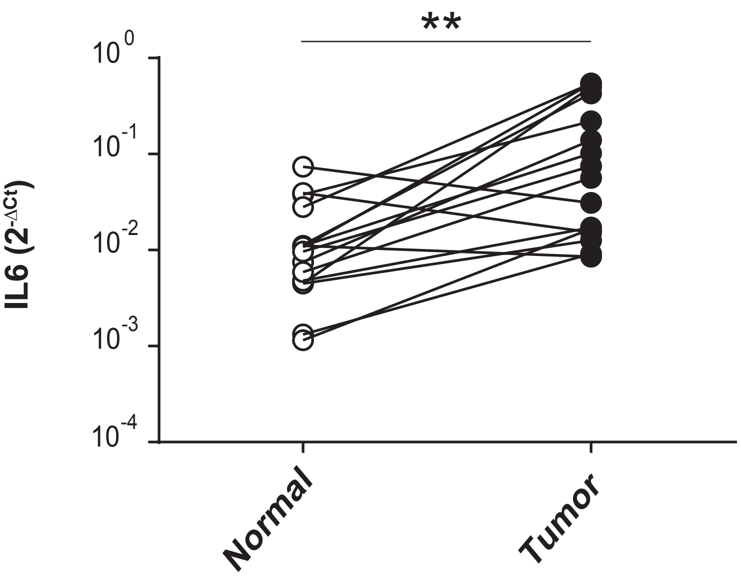

Supporting Information Figure 3. Expression of human *IL6* in colon cancer. Gene expression in tumor tissue and adjacent unaffected tissue from colon cancer patients (n=17) was analyzed by qPCR, run in duplicates, and determined using the $2^{-\Delta Ct}$ method with *HPRT1* as a reference gene. Wilcoxon matched-pairs signed rank test was used for statistical analysis (*P≤0.05, **P≤0.01, ***P ≤0.001 and ****P≤0.0001). Connecting lines show values from samples taken from the same individual.
