# Supplementary material for: Altered expression of Butyrophilin (BTN) and BTN‐like (BTNL) genes in intestinal inflammation and colon cancer
Source: Immun Inflamm Dis. 2016 Apr 1;4(2):191–200. doi: 10.1002/iid3.105 (PMC4879465; doi:10.1002/iid3.105)
Supplement: Supplementary file 2 — Table S1. Characteristics of the colon cancer patients included in the study. [file IID3-4-191-s002.pdf]

		Females	Males
**n**		7	10
**Age**		52-77	42-79
**Tumor location**	**ceacum**	-	2
	**ascending**	2	1
	**transverse**	1	2
	**descending**	1	1
	**sigmoid**	3	4
**Differentiation grade**	**high**	-	-
	**medium**	7	10
	**low**	-	-
	**mucinous**	-	-
**Tumor stage**	**T1**	-	-
	**T2**	-	1
	**T3**	4	4
	**T4**	3	5
**Lymph node spread**		6	6
**Distant metastases**		1	-

Supporting Information Table 1. Characteristics of the colon cancer patients included in the study.
