# Supplementary material for: Altered expression of Butyrophilin (BTN) and BTN‐like (BTNL) genes in intestinal inflammation and colon cancer
Source: Immun Inflamm Dis. 2016 Apr 1;4(2):191–200. doi: 10.1002/iid3.105 (PMC4879465; doi:10.1002/iid3.105)
Supplement: Supplementary file 3 — Table S2. Primer sequences. [file IID3-4-191-s003.pdf]

Gene	Primer sequence	
	Forward	Reverse
**Human**		
HPRT1	Primers were purchased from Applied Biosystems (Hs99999909_m1)	
BTN1A1	5´-ggatggaagctacgaagaagc-3´	5´-tgcatactgatgtgagggtca-3´
BTN2A1	5´-aggagaccagatttcgtttcct-3´	5´-agggcagcagctgattccat-3´
BTN2A2	5´-gaaggcaggtcctacgatga-3´	5´-tgggccttgatttcaatgag-3´
BTN3A1	5´-tcagaggggaatgctaagagg-3´	5´-caagtatggtgaccgaagaaga-3´
BTN3A2	5´-ctccaatgggaataccaagg-3´	5´-gggaacttgccattttcatcta-3´
BTN3A3	5´-actcaagtggaggaaaatccagt-3´	5´-tggcagatcccgcggctct-3´
BTNL2	5´-agaaggggtcggtcatcag-3´	5´-gctgtatatcttctcccactctgac-3´
BTNL3	5´-tcagtttctacgagctggtgtc-3´	5´-ccaaggcctggacaaactt-3´
BTNL8	5´-gctctcatgctcagtttggtt-3´	5´-gtctggcccaaacacctg-3´
BTNL9	5´-tcttgtcttcctcatgcacct-3´	5´-gcctagcaccttgacctctg-3´
**Murine**		
β-actin	5´-cttctttgcagctccttcgtt-3´	5´-aggagtccttctgacccatgc-3´
Btn1a1	5´-tactggccttaggatttctcacc-3´	5´-gacgtgaatcttccaatcgaact-3´
Btn2a2	5´-tggagacgaaccctcttacatg-3´	5´-cacatggacggcagtcaaatc-3´
Btnl1	5´-tgaccaggagaaatcgaagg-3´	5´-caccgagcaggaccaatagt-3´
Btnl2	5´-ttcacaatgccagaacttcg-3´	5´-ttccatctctgtccctccac-3´
Btnl4	5´-cattctcctcagagacccacacta -3´	5´-gagaggcctgagggaagaa-3´
Btnl6	5´-atccttggagatccacagtgaa-3´	5´-gggagagaccttgggaaaga-3´
Btnl9	5´-cccctttagagggaggtga-3´	5´-aatactgagaaatctgccatctgtc-3´

Supporting Information Table 2. Primer sequences.
